# Supplementary material for: Use of GPS to measure external load and estimate the incidence of muscle injuries in men’s football: A novel descriptive study
Source: PLoS One. 2022 Feb 4;17(2):e0263494. doi: 10.1371/journal.pone.0263494 (PMC8815982; doi:10.1371/journal.pone.0263494)
Supplement: S1 Appendix — (DOCX) [file pone.0263494.s001.docx]

S1 Appendix: Specific targets for each training day relative to the match

The -4MD sessions were designed to develop the players’ strength and power in tasks with limited spaces (Area: 30-80m^2^ per player, i.e., positional games (PG)5v5+2, 6v6+2 and small-sided games (SSG) 5v5v5, 6v6v6 with goalkeepers (GK)). The -3MD session has the closest context to that of the match, in that its objective is tactically prepare the players for the next match by having them perform tasks in larger spaces (Area: >125m^2^ per player, i.e., PG 8v8+2, 9v9+2 and 9v9 to 11v11 matches with GK), often nearing competition dimensions. The -2MD session focused on technical-tactical elements in small spaces (Area: 30-80m^2^ per player, i.e., PG 4v4+3, 5v5+2 and SSG 5v5v5 with GK) and with lower loads. The -1MD session was the final pre-competition session and involved activation-oriented tasks with reduced external load as the competition approached.
