# Supplementary material for: Use of GPS to measure external load and estimate the incidence of muscle injuries in men’s football: A novel descriptive study
Source: PLoS One. 2022 Feb 4;17(2):e0263494. doi: 10.1371/journal.pone.0263494 (PMC8815982; doi:10.1371/journal.pone.0263494)
Supplement: Attachment — Submitted filename: Comments_Mus Inj Incid_GPS_PLOS.docx [file pone.0263494.s002.docx]

Thank you for the opportunity to review the manuscript titled ‘Muscle injury according to exposure of time and global positioning satellite system metrics: A descriptive study from a senior men’s and academy team belonging to a Spanish professional club’. Clearly there has been substantial data collection and analysis for this paper.

General comments:

Throughout the manuscript there are formatting issues for example line 63 to 69 does not constitute a paragraph, similarly lines 71 to 73, 155 to 156, 242 to 246, 269 to 271, 290 to 298, 429 to 432,452 to 454, 503 to 513, 517 to 534 need to be formatted correctly into paragraphs. Unfortunately, this gives an untidy appearance to the reader. Most of the authors of this study I would assume their first language is not English. While I do empathise that writing in a non-native language is difficult for this study to reach publication it would need extensive re-writing and structuring.

Introduction would be better if this section provided a clear argument for the need to calculate injury incidence using GPS metrics. At the moment this is missing in my view. There little argument and this section is very descriptive. In general, the introduction is too long and needs work on the content and structure. For example, if the main aim of the paper is to use GPS metrics to calculate injury incidence then injury incidence (specifically muscle injury) and the limitations of how they are currently calculated should be addressed in details. Line 91 to 102 focuses solely on GPS might be better to alter the emphasis and provide an argument for calculating injury incidence using these metrics, so practitioners understand the risk associated with specific training aims i.e., greater distances covered increases muscle injury risk. Check formatting throughout 2-4 sentences does not justify a new paragraph. Overall this section needs to be re-wrote and structured.

Discussion, the title does not reflect the aims of the main content i.e., muscle injury according to GPS metrics is not discussed until the fourth section of the discussion. Again, the discussion is too long and would benefit from more concise writing. More detailed and structured discussion of your findings is required. The first finding that is discussed *‘all parameters of player external load (HMLD,HSR, PL and TD)  in volume and intensity are according the club structure plan of training-match day’*; suggesting this is the major/novel finding from your data. Surely the major/novel finding should be muscle injury incidence according to GPS metrics? The fact that volume and intensities are inline with the club training plan might offer some explanation as to why muscle injury incidence according to GPS metric did not differ between senior and academy players. Overall this section needs to be re-wrote and structured for example, Line 495: ‘most important finding’…it has not been discussed until page 4 of the discussion.

The title suggests the main aim of the study was to provide injury exposure using GPS metrics however, this is the second aim of the study according to line 124-128.

Abstract

Line 28 to 30: the phrase ‘to guide’ used twice within a single sentence.

Line 31 to 36: If the main aim of the paper is to describe external loads across one club this should be reflected in the title? If injury according GPS metrics is the main aim please re-order.

Line 34: muscle injuries – are these lower limbs? Might be better to specific here and also throughout the manuscript.

Line 37 to 40: Needs to be re-worded.

Line 38: Details such as age, height, weight and position would be useful for the reader.

Line 45 to 47: GPS metrics need to be ordered the same throughout the manuscript.

Line 56 to 58: Need to be re-worded. The novel finding here appears to be that higher intensity training day is associated with increased muscle injury.

Introduction

Line 66: Economic reference, no peer review paper?

Batten L Football injury index: English Premier League 2019 review. *Marsh JLT Specialty* 1-22, 2019.

Ekstrand J Keeping your top players on the pitch: the key to football medicine at a professional level. *British Journal of Sports Medicine* 47: 723-724, 2013.

Eliakim E, Morgulev E, Lidor R, Meckel Y Estimation of injury costs: financial damage of English Premier League teams’ underachievement due to injuries. *BMJ Open Sport Exerc Med* 6: e000675, 2020.

Hickey J, Shield AJ, Williams MD, Opar DA The financial cost of hamstring strain injuries in the Australian Football League. *British Journal of Sports Medicine* 48: 729-730, 2014.

Line 71 to 73: Is this meant to be here? Prior to this you describe injury consequences then after these lines you describe muscles injury. Line 71 to 73 disrupts the flow of your argument, and therefore, needs to be linked with pre and post sentences.

Line 76: ‘athletes?’ should this be footballers?

Line 76 to 81: This is too long and very hard to follow. Is it not possible to break this into 2 or 3 sentences?

Line 76 to 77: Starts sentence ‘With’ and then repeats ‘with the objective’…may be re-word?

Line 77 to 81: Too long for a single sentence and too many points. Might be better broken into 3 sentences 1 point per sentence.

Line 80: ‘also and careful and appropriate’…I am unsure what you are trying to say here, please re-word. Might simply be the case of a typo, but the sentence is unclear.

Line 88 to 89: Is there need for parentheses at the end of the sentence.

Line 91 to 95: Too long for a single sentence and too many points. Might be better broken into 3 sentences 1 point per sentence.

Line 102: Can you support this statement with a reference?

Line 106: ‘artefacts and artificial alterations’ could you provide a specific example for the reader.

Line 109: “Despite many publications’ could you provide specific examples of these publications for the reader.

Line 114: ‘incidence is typically calculated using..’ can you support this statement with a reference. You already mention this Line 71 to 73, can you see how this affects the flow for the reader? Each sentence should link.

Line 114: replace global with total?

Line 119: add ‘in’ after ‘training day’.

Line 120: To assist the reader it might be worth defining what is meant by category.

Line 124: This needs to be clearer for example ‘To investigate external loads and intensities of academy and professional footballers according to…’

Line 124 to 128: What is the main aim of this study is it to use GPS metrics to calculate injury incidence or explore external loads/intensities. This aim should be reflected in the title and also in the order of the aims.

Methods

Line 134 to 146: Start almost all sentences with ‘We’, might be better to selected different words.

Line 161: typo ‘agrouped’.

Line 165: (19,23). Remove the full stop.

Line 170-178: What is a larger space can you provide a diagram with distances i.e., 100x60 to 80x48m, competition dimension is a little vague the same for small spaces. Is it possible to define technical-tactical, activation- oriented tasks…again could you be specific player numbers would also be very useful. An overview of a typical training schedules and its contents would also help the reader.

Line 176: ‘wasn’t’ better to replace with ‘was not’

Line 176: ‘analized’ throughout the paper there are several different spellings of this word, please check and correct.

Line 183: ‘analysed’ consistent spelling of this word is required throughout this paper.

Line 192: Person’s correlation…is this reported correctly

Line 192 to 193: Intra and inter unit reliability would be useful here to show some data.

Line 199: This sentence may need re-wording

Line 223: Is there a reason for using the Hägglund et al paper and not the injury consensus?

Line 225: typo ‘te’

Line 240: Is this full training if so this would be better to clearly state.

Line 250: ‘We performed a descriptive study’ is this needed.

Line 262: IR could you please define what this means.

More details are required for how you calculated injury incidence with GPS metric. Again, this section is very long, but with some important information missing especially if the reader has no football background.

Results

This section is too long and needs to be concise, focus on the main findings.

Line 316: ’34 episodes of muscle injury’ may be better to include additional information such as location and severity. The injury consensus statement could be useful here.

Discussion

Start this section with a review of the aims of the study. Then in order of importance briefly present your findings. The sub-sections of the discussion should be structured in order of importance to assist the reader and also your argument.

Line 386 to 389: The training loads are in line with the club training plan? Was this the main aim of your paper, was this the main finding?? My opinion the higher muscle injury incidence on MD-3 is the novel finding – or not? Larger spaces and players number resulted in a higher muscle injury incidence (this novel), discuss why i.e., similar physical requirements to a match day, what are these requirements and why do they increase injury risk?

Line 390 to 392: Was injury higher in match day? This is not very clear, I would re-word this sentence, also you need a reference ‘like in previous studies’ such a statement requires support.

Line 393: ‘relevant differences’ I think this is not the correct wording

Line 404 to 414: The content here is very similar to that of line 169 to 174.

Line 404 to 422: Should this be the first sub-section. Again, the structure has to reflect your major/novel findings. In my view this is one of the least important findings of your study.

Line 411: ‘lower loads’ could you be more specific here total distance, HSR etc..

Line 413: ‘external loads’ do you mean all variables…. please specify.

For both these points might also be worth pointing the reader to the appropriate figures to support what you are saying.

Line 417: ‘Out with’ re-word

Line 417 to 420: Could these points be supported with a figure.

Line 426: ‘pro team’, you mean professional team needs to be consistent throughout the paper.

Line 429: typos ‘stuy’ ‘they’ ‘differenecs’

Line 431: Can you reference the past studies please.

Line 446: Di Salvo et al. no number

Line 453 to 454: Needs to be re-worded…’also’ might need to be added

Line 460 to 483: This should be the first section, if the paper is to reflect the title.

Line 496 and 471: Denominator is this correct word please check

Line 472 to 473: Both start with ‘We’ consider using a different word.

Line 473: ‘We really believe’ is this appropriate for a scientific journal?

Line 476: typo ‘Then seems that’ and ‘quantyfie’, check this line.

Line 490: ‘It has been shown that the injury incidence is higher when you play matches’. Replace ‘when you play matches’ with in competitive matches. Needs a reference to support your statement.

Line 490: Ekstrand 2011 no number.

Line 493: ‘We..we’. Better to re-word sentence.

Line 495: ‘most important finding’…it has not been discussed until page 4 of the discussion.

Line 509: What do these number represent, please be clear.

Line 518: What are the three variables

Line 520: ‘Mildfieldres’ replace with Midfielders

Line 524: typo ‘midfielders players’

Line 526 to 528: If your findings agree with Bacon et al. be clear and state. What is written is unclear.

Line 539 to 542: How does this impact on external validity. Does this paper have an advantage of a single doctor assessing injury? If so state this and support with a reference

Conclusion

The order of the conclusion is not consistent with the discussion.

Line 551: ‘This study present that’ word missing.

Line 556: ‘will have to be involved’ needs to be re-worded.

Contributors

No mention of author AM

References:

Number 5 needs capital letters replacing with lower case in the article title.

Number 11 ‘Owen2’

Number 13 needs capital letters replacing with lower case in the article title.

Number 17 needs capital letters replacing with lower case in the article title.

Number 27 needs capital letters replacing with lower case in the article title.

Number 29 please re-check

Number 30 ‘H??gglund’ Wald??n’

Number 37 needs capital letters replacing with lower case in the article title.
