# Supplementary material for: Use of GPS to measure external load and estimate the incidence of muscle injuries in men’s football: A novel descriptive study
Source: PLoS One. 2022 Feb 4;17(2):e0263494. doi: 10.1371/journal.pone.0263494 (PMC8815982; doi:10.1371/journal.pone.0263494)
Supplement: Attachment — Submitted filename: PLOSone_review.docx [file pone.0263494.s004.docx]

Thanks for the opportunity to review this paper. Again, it is clear to see that substantial effort has gone into the corrects however, there are still some issues that need to be addressed. I’m unsure if the conversation from a MS word document to a PDF is altering the format, but this does give the manuscript an untidy appearance (Line 126-129; 149-151). The entire article needs to be checked formatted correctly before submission.

Section feedback:-

Introduction

This section is too long (1011 words) and needs to be reduced by at least 50%. The main aim of this paper is ‘To investigate the external 140 load and intensities in players of a academy and professional football players according 141 to playing position and training day respect to match day’ yet there is little review of external load literature in the introduction.

Methods

Similar to the introduction the methods section is very long (1369 words) and would benefit from a significant reduction. While I empathise that the first language of authors is unlikely to be English, concise writing is required. Line 222-224 it states average physical parameters (volume and intensity) were calculated as a mean for all sessions, can you justify this method within the available literature?

Results

Again, this section is very long and could be improved by a reduction of words. To assist the reader rather than stating ‘showed very similar distributions’ please provide the data.

Discussion

Without doubt this section has been improved but the formatting is not to standard (e.g., line 415-418). However, the discussion is also very long and like the previous sections needs to reduced if it is to reach publication. More analysis of the findings are required, how does your main findings compare with the literature and if they are different why is that.

Specific Feedback

Line 68-69: Do clubs have their own economy? Better to re-write and use ‘economical consequences’

Line 75: Brackets next to brackets looks untidy “(up to 5 times higher ), (2,9–11)”

Line 80: Check full stop next to Thus

Line 93: Not very clear ‘if what has been planned is achieved or not’ ‘the training aim was’

Line 96-101: Very long for a single sentence hard to follow.

Line 106-111: Very long for a single sentence hard to follow.

Line 143: ‘day respect category’ - this is not clear

Line 144: ‘the season’ – a typical season?

Line 157: We

Line 159: We – consider re-wording.

Line 160: This

Line 162: This – consider re-wording.

Line 167: ‘Date’ – Data?

Line 167: ‘throght-out’ – typo?

Line 178: Brackets next to brackets looks untidy ‘(Youth (U18 and 179 U19) and Professional)’

Line 178-185: Too long and difficult to follow.

Line 187: ‘strength and power in tasks with limited spaces’ could you provide an example

Lone 189: ‘perform tasks in larger spaces’ could you provide an example i.e., 11v11-10v10

Line 191: ‘technical-tactical elements in small spaces’ again please provide an example

Line 193: ‘activation-oriented tasks’ again please provide the reader with an example

Line 203: was this reliability work done by your group? If not is there need to describe this, may just use the data and reference.

Line 206: The

Line 208: The – consider re-wording

Line 211: The - consider re-wording

Line 224-225: should the playing positions be capital letters?

Line 241: ‘We define an injury as 242 any injury’ – does not read clearly

Line 254: ‘eliminates interpersonal biases’ – can you support this with a reference?

Line 277: Again, player positions in capitals, is this required?

Line 302: ‘a.u’ – earlier written in capitals?

Line 316: ‘All four parameters showed very similar distributions’ - and the distributions were? Some data would be useful here?

Line 318: ‘varied according to’ – what was the variation? Data would be useful here.

Line 319: ‘pronounced for the HSR variable than for the other variables’- Data would be useful here.

Line 332: ‘similar distributions’ data would be good here

Line 333: ‘varied according to’ data would be good here

Line 336: ‘shown in Figure 1 above’ should this be here as this section relates to figure 2 – may be this needs to be moved up to figure 1 section.

Line 341: TL, but in Line 357 Time Loss??

Line 408-411: Your points here are not very clear.

Line 438-442: So these finding (Martin-Garcia et al) are from a different club or the same club? It is not clear.

Line 445-448: Not possible to discuss your findings with those in the literature? What is here provides the reader with will useful information.

Line 449-451: why could this be? Good point to discuss further – less tactical knowledge?

Line 452-456: Too many points for a single sentence.

Line 463: ‘Full back’s and Forward’ capitals again…are these required?

Line 463: ‘showed slightly higher behaviour’….is this really a behaviour?

Line 466: ‘and Di Salvo et al. (45) analysed the distance travelled by 300 elite players, 467 observed significant differences according to the playing position’ – this needs to be re-written.

Line 469: “Second-Strikers and Wide-Midfielders” – capitals?

Line 470: How does your analysis explain the differences here could you provide more information as to why?

Line 486: ‘a total IR 2.48 (1,37 injuries training and 8,7 match)’ – is this per 1000 hours? If so please state it clearly.

Line 488: Could you include injury burden?

Line 492: ‘Nofind big differences’ – Not clear and needs better wording.

Line 484-495: ‘to know what happen’ – not very clear and would need better wording.

Line 495: ‘Then seems’ – may be a word missing here

Line 512: ‘(8.70 vs 1.37/1000 hours, P \ .001)’ – needs a consistent approach throughout the paper.

Line 520: ‘any remarkable differences’ – what is remarkable ‘sigificant’

Line 524-526: ‘Injury incidences were higher in the professional category (IRTAE = 1.59; IRHMLD = 0.26; IRHSR =0.95; IRLoad =3.03; IRDistance = 4.10) compared to youth players (1.34; 0.21; 0.84; 2.51; 3.3) respectively’. – would this not be explained by more compliant muscle/tendon unit, reduced force capacity etc…of younger players??

Line 535-536: with a notable difference – could you be clearer here what is notable significant?

Line 542-543: Your data agrees with Bacon et al? could this not be signposted a little better?

Line 549: What 2 categories?

Line 557: Only one club – what are the specific limitation of this?

Line 557: ‘has been’ – was?

Line 565-568: instead of list all staff members would it not be better ‘those working in academies and professional teams to better understand…’. I believe this is a really important section, but the points are not clear and do not stand out.

Line 572: ‘and midfielders’ – been in capitals all the way through the paper?
