# Supplementary material for: Use of GPS to measure external load and estimate the incidence of muscle injuries in men’s football: A novel descriptive study
Source: PLoS One. 2022 Feb 4;17(2):e0263494. doi: 10.1371/journal.pone.0263494 (PMC8815982; doi:10.1371/journal.pone.0263494)
Supplement: Attachment — Submitted filename: Comments_R2.docx [file pone.0263494.s006.docx]

**TITLE:** **Descriptive analysis of GPS as exposure measures and its use in estimating the**

**muscle injury incidence from a senior men’s and academy football team of a Spanish**

**professional club**

This study investigates external load and injuries in two groups of football players from a professional football club

**MAJOR COMMENTS**

- There are many grammar and typing errors throughout the manuscript. The manuscript should be revised thoroughly. There are many English mistakes. In my opinion, there are mistakes from the beginning, even the title of the paper does not seem correct. A native English speaker should revise the manuscript.
- In the title and the manuscript, the authors state “senior men’s and academy football”, according to this it looks as senior players were male, but academy players were male and female? Please re-write the whole title
- There are many typing and English errors:

Line 15: Is it correctly written “Barcelonatech”? or should it be ‘BarcelonaTech’?

Line 6: Please correct the mistake: “National Institute of Physical Education fo Cataonia (INEFC) University of Barcelona, Spain”

Line 40: “ They were 30 professional male and 41 male youth academy” is not correct. Line 34 and 36: “according to categories/exposure” should be rewritten, it is not correct

Line 36-37: needs rewriting, probably it needs a comma.

Line 47-48: Should “Player load” and “Total distance” be written with capital letters as the rest of the nouns?

Line 68: “fitness (…) teams”?

Line 69: “*contemporary* training programs” Difficult to understand

Line 147: check and change “trainnig”

Line 335: “highes”?

Line 371: “In principle”?

[There are too many mistakes and errors for a reviewer to correct them all, please check carefully the manuscript. It is not acceptable as it is now]

ABSTRACT

- In the abstract, the authors say in line 37 that this is a descriptive epidemiological study, however I think that this study is descriptive; but it is not epidemiological. Consequently, this word should be replaced in the *key words list*. The same applies for line 134.
- Line 36: What do the authors mean with the term “external load exposure”?
- Line 43-46. Rewrite this sentence, it is not correct. Moreover, what does “and preseason sessions” mean? This is not clear. The authors have not explained what “sessions” means. Probably the authors could separate the sentence.
- Line 39-41: These two sentences could joined
- Line 46: eliminate “the”
- Line 52: “training session/MD” has not been specified beforehand.
- Lines 52-55: These two sentences could joined, because they repeat information. The abstract should be clear and concise.
- Line 56: The authors state “…exposure presented a similar profile”. It is difficult to understand what the authors refer to. They should clarify what are they comparing.
- Lines 58-61: On one hand, the word “presents” should be changed. On the other, it is difficult to understand the whole meaning of these sentences.
- A conclusion is lacking at the end of the abstract
- In my opinion, the word “epidemiology” should be removed from the key words as this is not an epidemiology study.

INTRODUCTION

- Lines 70-72. In correct. Perhaps remove the word “while”?
- Lines 73. What do the authors mean with the word “normal”?
- Lines 78-83. Rewrite and use commas to separate ideas.
- Line 91. Please amend this: “hours(9-12 (13,14)”
- Line 93: Please write “mesures” correctly
- Line 102: Please correct “a academy”
- Lines 105-206: This sentence should be corrected. Please change “the training respect category…season”

METHODS

- Lines 108 and 110: Remove :
- Line 110: change capital letters
- Line 118: rewrite “categorie”
- Line 118: Please rewrite: “plays the 2^nd^…” (“in” is lacking, perhaps)
- Information about the Ethics Committee has been explained in page 6 and 10, please delete one.
- Line 129 and 130: Replace ; by : after the teams.
- The methodology should be clearer about the registered days. Did the authors separate season and preseason training days? If so, it should be stated clearer. Moreover, they should also clarify if footballers played any matches during the preseason (any friendly matches?).
- Line 147: What does the subtitle mean? It is difficult to understand because the English is not correct
- Line 150: missing space in “Intra-and”
- Line 158: Check the verbs (have been studied?)
- Line 158: Do they only refer to the training sessions or also the match? Please clarify
- Line 159: remove the symbol [
- Line 160: THe authors should choose the format for the units and write them all in the same staile (W/kg or km·h^-1^ or units*min)
- Lines 158-167: Check the capital letters, it would be better to remove them all
- The methods section have improved now. Information of Table 1 should referenced (i.e. name the papers)
- Line 167. Please check the sentence: “These parameters…Table1”
- Line 169: Table 1: 5 m/s^2^ 🡪 this unit means that this is an acceleration, therefore it is not possible to run at a constant speed of 5 m/s^2^, please amend this.
- Table 1: Authors should write the abbreviations of the table
- Table 1: The authors state that the HSR is the distance covered above 21 km/h, however they do not explain the reason for that. Moreover, it is questionable that 21km/h is high speed running for all players, this would suggest that all players have the same running speed. The authors should explain the reason for choosing 21 km/h instead of 20 km/h or other velocity.
- Line 163: Why are the volume and intensity called “physical parameters”? Moreover, the authors use the term “physical intensity parameters” (line 246) which is an unusual term
- Line 174: There is a space and a . missing after (17)
- Line 177: The verbs should be in past tense
- Line 182: It is difficult to understand why the authors refer to injuries as “sports-related lower limb injuries”.
- Line 187: The authors state “…we used during the first week of the season” which is difficult to understand. Does it mean that only pre-seasonal injuries were classified according to the severity? Please clarify
- Lines 200-202. This information is redundant (see lines 158-163).
- An statistical analysis should be performed in order to compare load and injuries amongst groups (youth vs senior, positions etc). In this line, sometimes authors assume that two values are different, but because there is no real analysis it is not possible to know if the difference is significant. For example, line 281: is 1.59 larger than 1.34? How large is this difference?

RESULTS

- The quality of the figures is poor, as a consequence, it is difficult to observe the results
- Line 225: Remove “the” figure…..
- Line 231: the authors wrote “Box plots without represent outliers…” which in my opinion it is not correctly written
- Figure 1: What does “Distance” mean? Total distance? Please amend
- Line 236: Authors wrote “The global median (IQR)” however, 1) they should clarify what IQR means (interquartile range?), 2) in the statistical analysis it is not mentioned and 3) in the statistical analysis the authors state that they calculated averages, not medians. Please clarify.
- Line 237-238, 243 and 255-256: Some of the units are missing
- Line 243: gain authors mention median values, however in the statistical analysis they mention that the calculated the mean (line 195). Please clarify
- Line 246-247: Please be careful with the extra spaces between words and “min-“
- Line 255: What does “The global median” mean? Please rewrite
- Lines 242 and 257-259: Authors state that the values were higher or lower, it is necessary to apply the corresponding statistical analysis in order to ascertain if the differences are statistically sound and to quantify the differences. This analysis would reinforce the results and the power of the paper.
- Table 2: remove the title of the table form the table, they should be apart
- Table 2: Remove quotation marks
- Table 2: The number of injuries was very low, besides medical attention were included (no time loss injuries) in comparison to other studies i.e. Ekstrand J, Hägglund M, Waldén M. Epidemiology of muscle injuries in professional football (soccer). Am J Sports Med. 2011;39(6):1226-32. doi: 10.1177/0363546510395879. This issue should be discussed accordingly
- Line 281: Authors state that TL injury incidence was higher in the professional players vs youth players (1.59 vs 1.34), however, due to the fact that there is no statistical analysis this cannot be corroborated. The same applies to other results i.e. 0.25 vs 0.20 (line 289) what is the real relevance of this? Are they really “different”?. A proper statistical analysis would help to this.

DISCUSSION

- The discussion should start with an “introductory paragraph of the discussion section) including a summary paragraph and the objectives of the study.
- The discussion section is poor. Authors should discuss their results and explain them, giving reasons for it. In addition, they should compare to other studies.
- Paragraphs and sentences should follow a flow of results - ideas – comparison to other studies – discussion of the similarities/disparities….
- Practical applications: This paragraph should be rewritten in order to better clarify the real application of this study.
- Line 340: Please correct the sentence (professional and youth players?)
- Line 341-344: What is the reason for these sentences? It seems that this is an explanation for a result? Perhaps place it after the next paragraph?
- Line 345: The authors state that results of the external load was similar between professional and youth players; nevertheless, due to the low quality of the graphs it is not possible to see this results, there are no results written in the results section and there is no statistical analysis to confirm the similarities or the differences in the results.

Line 345-346: The authors explain their results but include an external reference (21). Please amend this incongruence

- Lines 353-363: check the format of the references and correct them. Check the English.
- Line 358: Difficult to understand “467 observed”
- Line 362. Check the verb tense. Also, link better both paragraphs.
- Line 368: This subtitle is not correct. Differences?
- Line 373: Correct the verb. Correct the term “what happens” it is very colloquial
- Lines 370-379: These are results; authors should avoid repeating results, unless it is necessary and discuss them, find the relevance and the meaning of the results and compare them to other authors.
- Lines 383-387: These are results again. The authors should mention the reasons for these results and the relevance
- Lines 388-391: There is an incongruence: the same training methodology leads to a difference in the injury incidence? Please explain
- Practical applications: Authors should rewrite these applications, in this version the applicability of the study is missing
- Conclusions: this paragraph is a summary of the results. Please rewrite the conclusions of the study

OVERALL COMMENTS

- The quality of the graphs is poor. It is very difficult to read the numbers and the letters
- There are many errors throughout the manuscript
- Check all the References: there are many mistakes, for example ref number 6, 11 (capital letters), 14 etc; remove [internet] and so on.
