# Supplementary material for: Use of GPS to measure external load and estimate the incidence of muscle injuries in men’s football: A novel descriptive study
Source: PLoS One. 2022 Feb 4;17(2):e0263494. doi: 10.1371/journal.pone.0263494 (PMC8815982; doi:10.1371/journal.pone.0263494)
Supplement: Attachment — Submitted filename: Review - Descriptive analysis of GPS as exposure measures and its use in estimating the muscle injury incidence from a senior men.docx [file pone.0263494.s007.docx]

Descriptive analysis of GPS as exposure measures and its use in estimating the muscle injury incidence from a senior men’s and academy football team of a Spanish professional club

Firstly, I apologies for the slow response this was due to unforeseen circumstances.

Thanks for allowing me to review this article. Again, you can see the effort that author have ‘put in’ to developing this manuscript. Peer review is never an easy process more so when English is not your first language. I have given some points for consideration concerning the introduction and discussion. If the information is presented to a high-level this could change the way injury incidence is calculated in the literature moving forward. However, it is going to require substantial re-working. This is the third time I have reviewed this paper and the same formatting issues are still present for example…

Line 121-125; Line 129-133; Line 186-189; Line 200-203; Line 212-214

Line 236-238; Line 240-244; Line 246-247; Line 255 to 256; Line 257-261

Single sentences should not form a new paragraph. Although it is possible that this is happening when converting to PDF, but checking the PDF before submission would help.

Graphs are unclear on my laptop, but again I’m not sure of this is a issue with conversion across platforms or OS systems.

As I read this paper the aims are:-

1….’to investigate the external load and intensities in players of a academy and professional football players according to playing position and training day respect to match day’

2…..’to evaluate incidence rates of sports-related lower limb muscle injuries according to exposure time, HMLD exposure and HSR exposure taking into account the training respect category, positions and match day during a typical season’.

Aim number 1 is the most important…if this is not the case then please re-structure your aims and make it clear.

The introduction should provide a clear and targeted argument for the requirement of external load and intensities in youth and senior players according to playing position and training day by focusing on the current lack of information. While also highlighting the benefits of muscle injury incidence rates being reported using GPS metric instead of the widely accepted 1000 hours. A small section of the introduction does discuss injury incidence but this can be done in greater detail. In my view the argument within your introduction could be clearer.

Potential Introduction structure

+Game/training demands using GPS metrics

+ Few studies provide information on youth and senior players training and playing using the same training/playing methodology.

+Injury incidence rates training and games youth and senior

+How and why 1000hours lacks context

+GPS metrics go some way to providing some context; yet never used to calculate injury incidence

Discussion

The separate headings assist the reader which is a positive. However, there is a lack of critical analysis of the results and also comparisons with previous research could be presented better. For example, Line 358-361 is ‘list like’ and these studies presented in this section could be written more concisely “Past research has identified that defenders cover less high intensity distances compared to other outfield positions (23, 24, 25). In contrast, we found that forwards and full backs covered greater HSR distances compared to other positions. These differences might be explained by tactical roles of full back and forwards, which are unique to each clubs’ playing style. The players used in this study were encouraged by the technical staff to….. which likely resulted in greater HSR distances. Moreover, analysing match data relative to playing minutes could further explain the differences between the findings of the current study and previous work”.

It is my view that every section in the discussion would benefit greatly from more critical analysis and concise writing. Of course, is always easy when (1) I speak English as a first language (2) I have hindsight on my side and likely more time to analyse what you have written.

Practice applications

This section is one of the most important within this manuscript , yet there is little analysis. Several of the authors are high-level applied practitioners working at the highest level of sport and it would greatly benefit this paper if more attention is paid to this section.

Introduction

Line 68-70 Consequently a focus for fitness and medical teams is to design contemporary training programmes with the objective of matching the game demands. May be use

*in line with game tasks and demands* to replace the words highlighted in red

Line 71-72 focusing on matching (aligning?) these game demands might paradoxically (?) also increase the risk of muscle injury during training. Can you support this statement? Matching is not a scientific word and paradoxically is there actual need for this word?

Line 72-74 Needs to be re-phrased ‘of types of drills’

Line 85-87 Unsure what the point you’re trying to say here. ‘Despite many publications in the area’ what area do you mean? ‘many publications’ you have only provided 1-reference?

Line 89-90 ‘Football injury GPS studies typically use an overall match and training injury rate’ This is unclear what you are saying here.

Methods

Line 121-125; Line 129-133; Line 186-189; Line 200-203; Line 212-214 – Are you sure these have enough content to be standalone paragraphs?

Line 135 ‘We analysed all weeks of…*We analysed all training weeks*

Line 137 Can you start a sentence with +1MD?

Line 139 ‘(these players performed recovery tasks)’ and these tasks are…more details needed

Line 140 ‘(these players performed compensatory load tasks)’ and these tasks are…more details needed

Line 149 ‘(Realtrack….there is no closing bracket

Line 150-151 (ICC)…can you have brackets within brackets?

Line 153 (version 927) can you have brackets within brackets?

Line 175 UEFA (17) – no full stop here…I think one is required

Line 165-179 sentence is very long and difficult to understand. Needs to be rephrased and divided into 2 or 3 separate sentences.

Line 185 ‘RTP’…this has not been defined, please define.

Line 193-194 While the content is correct, could it be rephrased and simplified so that it’s easily understood by readers with a limited background in statistics.

Line 196 ‘stratified’ would sub-group be a better phrase since it is easier to understand?

Line 216-219 Please forgive me if I am incorrect but this reads the same as line 123-125, its as if it’s been cut and pasted.

Results

Line 236-238; Line 240-244; Line 246-247; Line 255 to 256; Line 257-261 – Are you sure these have enough content to be standalone paragraphs? I think some of these can be combined or am I reading this incorrectly?

Line 257-261 This reads the same as Line 240-244. One of these sections needs to be rephrased.

Line 273-274; Line 292; Line 303; Line 313; Line 323 – brackets next to brackets…these sections should be re-formatted.

Line 281 ‘than in the’ change to compared to?

Line 287 ‘incidence by per… ‘I think you need remove ‘by’?

Discussion

Line 341-344; Line 345-349; Line 362-363; Line 383-387; Line 388-391 – again is there enough content here to create separate paragraphs?

Aim number 1….’to investigate the external load and intensities in players of a academy and professional football players according to playing position and training day respect to match day’

Aim number 2…..’to evaluate incidence rates of sports-related lower limb muscle injuries according to exposure time, HMLD exposure and HSR exposure taking into account the training respect category, positions and match day during a typical season’.

If these are the aims of this manuscript then your discussion should follow in this organisation. Line 332-337 should follow this structure.

Line 341 and Line 345 and 353 ‘In our’ a little repetitive may be change one of these.

Line 341 “in our football club’ what you write is correct but I am not sure such a statement belongs in a scientific journal.

Line 344 Great content but needs to rephrased.

Line 347-349 Needs to be rephrased

Line 355 ‘showed slightly higher values’…could you direct the reader to this data using a table or figure?

Line 355-356 ‘Our results are different from other authors’….needs rephrasing

Line 357-361 Valid points but need to be written in a concise manner that links together. At the moment they read like separate statements.

Line 390-391 You provide the same explanation twice (line 348-349), so what are the underpinning factors for this? Can you go into more details and provide some of the key characteristics of this methodology?

Limitations

Line 411-412 You correctly highlight a small sample size, single club and one season, but could you not give more information as to how these impact on your data – producing a source of bias, lacking external validity etc….

Line 414 ‘Future studies...’ I feel you could be more targeted here and give specific rather than generalising.

Line 415-416 ‘to be compared with the results of our work….’ Are you trying to say that your findings need to be validated by further research?
