# Supplementary material for: Use of GPS to measure external load and estimate the incidence of muscle injuries in men’s football: A novel descriptive study
Source: PLoS One. 2022 Feb 4;17(2):e0263494. doi: 10.1371/journal.pone.0263494 (PMC8815982; doi:10.1371/journal.pone.0263494)
Supplement: Attachment — Submitted filename: Reply_comments_reviewers_final.docx [file pone.0263494.s008.docx]

**Reviewer 1**

Descriptive analysis of GPS as exposure measures and its use in estimating the muscle injury incidence from a senior men’s and academy football team of a Spanish professional club

Firstly, I apologies for the slow response this was due to unforeseen circumstances.

Thanks for allowing me to review this article. Again, you can see the effort that author have ‘put in’ to developing this manuscript. Peer review is never an easy process more so when English is not your first language. I have given some points for consideration concerning the introduction and discussion. If the information is presented to a high-level this could change the way injury incidence is calculated in the literature moving forward. However, it is going to require substantial re-working. This is the third time I have reviewed this paper and the same formatting issues are still present for example…

**Thank you for your comment. We have carefully read the comments and suggestions that have certainly improved the manuscript. Additionally, a list of changes and answers to the reviewers’ comments has been submitted.**

Line 121-125; Line 129-133; Line 186-189; Line 200-203; Line 212-214

Line 236-238; Line 240-244; Line 246-247; Line 255 to 256; Line 257-261

**Done. Thank you**

Single sentences should not form a new paragraph. Although it is possible that this is happening when converting to PDF, but checking the PDF before submission would help.

Graphs are unclear on my laptop, but again I’m not sure of this is a issue with conversion across platforms or OS systems.

**We have followed the Figure File Requirements (https://journals.plos.org/plosone/s/figures#loc-figure-preparation-checklist) of the Plos and we haven’t had any problems when submitting it. It may be due to OS systems. However, we can ask the production team about the figures if the manuscript is finally accepted.**

As I read this paper the aims are:-

1….’to investigate the external load and intensities in players of a academy and professional football players according to playing position and training day respect to match day’

2…..’to evaluate incidence rates of sports-related lower limb muscle injuries according to exposure time, HMLD exposure and HSR exposure taking into account the training respect category, positions and match day during a typical season’.

Aim number 1 is the most important…if this is not the case then please re-structure your aims and make it clear.

**Thanks for your comment. When we chose our objectives, we decided to write them down in a chronological order of analysis, not in order of importance. That’s why, for us, the most important objective was number 2. However, after reading your comment we have rephrased this part of the section and we now have only one objective.**

The introduction should provide a clear and targeted argument for the requirement of external load and intensities in youth and senior players according to playing position and training day by focusing on the current lack of information. While also highlighting the benefits of muscle injury incidence rates being reported using GPS metric instead of the widely accepted 1000 hours. A small section of the introduction does discuss injury incidence but this can be done in greater detail. In my view the argument within your introduction could be clearer.

Potential Introduction structure

+Game/training demands using GPS metrics

+ Few studies provide information on youth and senior players training and playing using the same training/playing methodology.

+Injury incidence rates training and games youth and senior

+How and why 1000hours lacks context

+GPS metrics go some way to providing some context; yet never used to calculate injury incidence

Discussion

The separate headings assist the reader which is a positive. However, there is a lack of critical analysis of the results and also comparisons with previous research could be presented better. For example, Line 358-361 is ‘list like’ and these studies presented in this section could be written more concisely “Past research has identified that defenders cover less high intensity distances compared to other outfield positions (23, 24, 25). In contrast, we found that forwards and full backs covered greater HSR distances compared to other positions. These differences might be explained by tactical roles of full back and forwards, which are unique to each clubs’ playing style. The players used in this study were encouraged by the technical staff to….. which likely resulted in greater HSR distances. Moreover, analysing match data relative to playing minutes could further explain the differences between the findings of the current study and previous work”.

**Thank you for your suggestion. We have improved this section.**

It is my view that every section in the discussion would benefit greatly from more critical analysis and concise writing. Of course, is always easy when (1) I speak English as a first language (2) I have hindsight on my side and likely more time to analyse what you have written.

**Thank you. A native English speaker has reviewed the manuscript.**

Practice applications

This section is one of the most important within this manuscript , yet there is little analysis. Several of the authors are high-level applied practitioners working at the highest level of sport and it would greatly benefit this paper if more attention is paid to this section.

**Thank you for your suggestion. We have improved this section.**

Introduction

Line 68-70 Consequently a focus for fitness and medical teams is to design contemporary training programmes with the objective of matching the game demands. May be use

*in line with game tasks and demands* to replace the words highlighted in red

Line 71-72 focusing on matching (aligning?) these game demands might paradoxically (?) also increase the risk of muscle injury during training. Can you support this statement? Matching is not a scientific word and paradoxically is there actual need for this word?

Line 72-74 Needs to be re-phrased ‘of types of drills’

Line 85-87 Unsure what the point you’re trying to say here. ‘Despite many publications in the area’ what area do you mean? ‘many publications’ you have only provided 1-reference?

Line 89-90 ‘Football injury GPS studies typically use an overall match and training injury rate’ This is unclear what you are saying here.

**Thank you for your comments that really improves the section. We have tried to improve the introduction section.**

Methods

Line 121-125; Line 129-133; Line 186-189; Line 200-203; Line 212-214 – Are you sure these have enough content to be standalone paragraphs?

**Done. Thank you**

Line 135 ‘We analysed all weeks of…*We analysed all training weeks*

**Done. Thank you**

Line 137 Can you start a sentence with +1MD?

**Done. Thank you**

Line 139 ‘(these players performed recovery tasks)’ and these tasks are…more details needed

**Done. Thank you**

Line 140 ‘(these players performed compensatory load tasks)’ and these tasks are…more details needed

**Done. Thank you**

Line 149 ‘(Realtrack….there is no closing bracket

**Done. Thank you**

Line 150-151 (ICC)…can you have brackets within brackets?

**Done. Thank you**

Line 153 (version 927) can you have brackets within brackets?

**Done. Thank you**

Line 175 UEFA (17) – no full stop here…I think one is required

**Done. Thank you**

Line 165-179 sentence is very long and difficult to understand. Needs to be rephrased and divided into 2 or 3 separate sentences.

**Done. Thank you**

Line 185 ‘RTP’…this has not been defined, please define.

**Done. Thank you**

Line 193-194 While the content is correct, could it be rephrased and simplified so that it’s easily understood by readers with a limited background in statistics.

**Done. Thank you**

Line 196 ‘stratified’ would sub-group be a better phrase since it is easier to understand?

**Done. Thank you**

Line 216-219 Please forgive me if I am incorrect but this reads the same as line 123-125, its as if it’s been cut and pasted.

**Done. Thank you**

Results

Line 236-238; Line 240-244; Line 246-247; Line 255 to 256; Line 257-261 – Are you sure these have enough content to be standalone paragraphs? I think some of these can be combined or am I reading this incorrectly?

**Done. Thank you**

Line 257-261 This reads the same as Line 240-244. One of these sections needs to be rephrased.

**Done. Thank you**

Line 273-274; Line 292; Line 303; Line 313; Line 323 – brackets next to brackets…these sections should be re-formatted.

**Done. Thank you**

Line 281 ‘than in the’ change to compared to?

**Done. Thank you**

Line 287 ‘incidence by per… ‘I think you need remove ‘by’?

**Done. Thank you**

Discussion

Line 341-344; Line 345-349; Line 362-363; Line 383-387; Line 388-391 – again is there enough content here to create separate paragraphs?

**Done. Thank you**

Aim number 1….’to investigate the external load and intensities in players of a academy and professional football players according to playing position and training day respect to match day’

Aim number 2…..’to evaluate incidence rates of sports-related lower limb muscle injuries according to exposure time, HMLD exposure and HSR exposure taking into account the training respect category, positions and match day during a typical season’.

If these are the aims of this manuscript then your discussion should follow in this organisation. Line 332-337 should follow this structure.

**Done. Thank you**

Line 341 and Line 345 and 353 ‘In our’ a little repetitive may be change one of these.

**Done. Thank you**

Line 341 “in our football club’ what you write is correct but I am not sure such a statement belongs in a scientific journal.

**Done. Thank you**

Line 344 Great content but needs to rephrased.

**Done. Thank you**

Line 347-349 Needs to be rephrased

**Done. Thank you**

Line 355 ‘showed slightly higher values’…could you direct the reader to this data using a table or figure?

**Done. Thank you**

Line 355-356 ‘Our results are different from other authors’….needs rephrasing

**Done. Thank you**

Line 357-361 Valid points but need to be written in a concise manner that links together. At the moment they read like separate statements.

**Done. Thank you**

Line 390-391 You provide the same explanation twice (line 348-349), so what are the underpinning factors for this? Can you go into more details and provide some of the key characteristics of this methodology?

**Done. Thank you**

Limitations

Line 411-412 You correctly highlight a small sample size, single club and one season, but could you not give more information as to how these impact on your data – producing a source of bias, lacking external validity etc….

Line 414 ‘Future studies...’ I feel you could be more targeted here and give specific rather than generalising.

**Done. Thank you**

Line 415-416 ‘to be compared with the results of our work….’ Are you trying to say that your findings need to be validated by further research?

**Done. Thank you**

**Reviewer 2:**

**TITLE:** **Descriptive analysis of GPS as exposure measures and its use in estimating the**

**muscle injury incidence from a senior men’s and academy football team of a Spanish**

**professional club**

This study investigates external load and injuries in two groups of football players from a professional football club

**MAJOR COMMENTS**

- There are many grammar and typing errors throughout the manuscript. The manuscript should be revised thoroughly. There are many English mistakes. In my opinion, there are mistakes from the beginning, even the title of the paper does not seem correct. A native English speaker should revise the manuscript.

**Done. Thank you. A native English speaker has reviewed the manuscript.**

- In the title and the manuscript, the authors state “senior men’s and academy football”, according to this it looks as senior players were male, but academy players were male and female? Please re-write the whole title

**Done. Thanks for your comment. We have rephrased the title. We think the new title is more	accurate.**

- There are many typing and English errors:

Line 15: Is it correctly written “Barcelonatech”? or should it be ‘BarcelonaTech’?

**Done. Thank you**

Line 6: Please correct the mistake: “National Institute of Physical Education fo Cataonia (INEFC) University of Barcelona, Spain”

**Done. Thank you**

Line 40: “ They were 30 professional male and 41 male youth academy” is not correct.

**Done. Thank you**

Line 34 and 36: “according to categories/exposure” should be rewritten, it is not correct

**Done. Thank you**

Line 36-37: needs rewriting, probably it needs a comma.

**Done. Thank you**

Line 47-48: Should “Player load” and “Total distance” be written with capital letters as the rest of the nouns?

**Done. Thank you**

Line 68: “fitness (…) teams”?

**Done. Thank you**

Line 69: “*contemporary* training programs” Difficult to understand

**Done. Thank you**

Line 147: check and change “trainnig”

**Done. Thank you**

Line 335: “highes”?

**Done. Thank you**

Line 371: “In principle”?

**Done. Thank you**

[There are too many mistakes and errors for a reviewer to correct them all, please check carefully the manuscript. It is not acceptable as it is now]

ABSTRACT

- In the abstract, the authors say in line 37 that this is a descriptive epidemiological study, however I think that this study is descriptive; but it is not epidemiological. Consequently, this word should be replaced in the *key words list*. The same applies for line 134.

**Done. Thank you**

- Line 36: What do the authors mean with the term “external load exposure”?

**Done. Thank you**

- Line 43-46. Rewrite this sentence, it is not correct. Moreover, what does “and preseason sessions” mean? This is not clear. The authors have not explained what “sessions” means. Probably the authors could separate the sentence.

**Done. Thank you**

- Line 39-41: These two sentences could joined

**Done. Thank you**

- Line 46: eliminate “the”

**Done. Thank you**

- Line 52: “training session/MD” has not been specified beforehand.

**Done. Thank you**

- Lines 52-55: These two sentences could joined, because they repeat information. The abstract should be clear and concise.
  - Line 52 we sepeak about training load
  - Line 55 we sepaak about injuyri incidence.
  - Must we joined?

**Done. Thank you**

- Line 56: The authors state “…exposure presented a similar profile”. It is difficult to understand what the authors refer to. They should clarify what are they comparing.

**Done. Thank you**

- Lines 58-61: On one hand, the word “presents” should be changed. On the other, it is difficult to understand the whole meaning of these sentences.

**Done. Thank you**

- A conclusion is lacking at the end of the abstract

**Done. Thank you**

- In my opinion, the word “epidemiology” should be removed from the key words as this is not an epidemiology study.

**Done. Thank you**

INTRODUCTION

- Lines 70-72. In correct. Perhaps remove the word “while”?

**Done. Thank you**

- Lines 73. What do the authors mean with the word “normal”?

**Done. Thank you**

- Lines 78-83. Rewrite and use commas to separate ideas.

**Done. Thank you**

- Line 91. Please amend this: “hours(9-12 (13,14)”

**Done. Thank you**

- Line 93: Please write “mesures” correctly

**Done. Thank you**

- Line 102: Please correct “a academy”

**Done. Thank you**

- Lines 105-206: This sentence should be corrected. Please change “the training respect category…season”

**Done. Thank you**

METHODS

- Lines 108 and 110: Remove :

**Done. Thank you**

- Line 110: change capital letters

**Done. Thank you**

- Line 118: rewrite “categorie”

**Done. Thank you**

- Line 118: Please rewrite: “plays the 2^nd^…” (“in” is lacking, perhaps)

**Done. Thank you**

- Information about the Ethics Committee has been explained in page 6 and 10, please delete one.

**Done. Thank you**

- Line 129 and 130: Replace ; by : after the teams.

**Done. Thank you**

- The methodology should be clearer about the registered days. Did the authors separate season and preseason training days? If so, it should be stated clearer. Moreover, they should also clarify if footballers played any matches during the preseason (any friendly matches?).

**Done. Thank you**

- Line 147: What does the subtitle mean? It is difficult to understand because the English is not correct

**Done. Thank you**

- Line 150: missing space in “Intra-and”

**Done. Thank you**

- Line 158: Check the verbs (have been studied?)

**Done. Thank you**

- Line 158: Do they only refer to the training sessions or also the match? Please clarify

**Done. Thank you**

- Line 159: remove the symbol [

**Done. Thank you**

- Line 160: THe authors should choose the format for the units and write them all in the same staile (W/kg or km·h^-1^ or units*min)

**Done. Thank you**

- Lines 158-167: Check the capital letters, it would be better to remove them all

**Done. Thank you**

- The methods section have improved now. Information of Table 1 should referenced (i.e. name the papers)

**Done. Thank you**

- Line 167. Please check the sentence: “These parameters…Table1”

**Done. Thank you**

- Line 169: Table 1: 5 m/s^2^ 🡪 this unit means that this is an acceleration, therefore it is not possible to run at a constant speed of 5 m/s^2^, please amend this.

**Done. Thank you**

- Table 1: Authors should write the abbreviations of the table

**Done. Thank you**

- Table 1: The authors state that the HSR is the distance covered above 21 km/h, however they do not explain the reason for that. Moreover, it is questionable that 21km/h is high speed running for all players, this would suggest that all players have the same running speed. The authors should explain the reason for choosing 21 km/h instead of 20 km/h or other velocity.

**Thank you very much for your contribution. The registration of previous sessions with other GPS system is a decision of the club.**

- Line 163: Why are the volume and intensity called “physical parameters”? Moreover, the authors use the term “physical intensity parameters” (line 246) which is an unusual term.

**Done. Thank you**

- Line 174: There is a space and a . missing after (17)

**Done. Thank you**

- Line 177: The verbs should be in past tense

**Done. Thank you**

- Line 182: It is difficult to understand why the authors refer to injuries as “sports-related lower limb injuries”.

**Done. Thank you**

- Line 187: The authors state “…we used during the first week of the season” which is difficult to understand. Does it mean that only pre-seasonal injuries were classified according to the severity? Please clarify

**Done. Thank you**

- Lines 200-202. This information is redundant (see lines 158-163).
- An statistical analysis should be performed in order to compare load and injuries amongst groups (youth vs senior, positions etc). In this line, sometimes authors assume that two values are different, but because there is no real analysis it is not possible to know if the difference is significant. For example, line 281: is 1.59 larger than 1.34? How large is this difference?

**Thanks. The purpose of our article is merely descriptive. That is, we are just describing our sample doing comparisons between some observed indicators. As you point out, if we would want to do inference about these comparisons, we should perform some statistical analysis taking into account the intra-individual correlation for each player. Furthermore, the external validation of the inference made to players from other teams would be compromised by having a sample of players from the same club. Therefore, our aim is to show a first descriptive picture of some relevant indicators.**

RESULTS

- The quality of the figures is poor, as a consequence, it is difficult to observe the results

**Done. Thank you**

- Line 225: Remove “the” figure…..

**Done. Thank you**

- Line 231: the authors wrote “Box plots without represent outliers…” which in my opinion it is not correctly written

**Done. Thank you**

- Figure 1: What does “Distance” mean? Total distance? Please amend

**Done. Thank you**

- Line 236: Authors wrote “The global median (IQR)” however, 1) they should clarify what IQR means (interquartile range?), 2) in the statistical analysis it is not mentioned and 3) in the statistical analysis the authors state that they calculated averages, not medians. Please clarify.

**Thank you for your suggestion. The Box-plots show the median and the interquartile range (IQR) of the averages of each player along all the season. We have included and clarified it as you suggested in the Statistical Analysis section.**

- Line 237-238, 243 and 255-256: Some of the units are missing

**Done. Thank you**

- Line 243: gain authors mention median values, however in the statistical analysis they mention that the calculated the mean (line 195). Please clarify

**Thanks. As it was written before, we have included and clarified it as you suggested in the Statistical Analysis section. First, we calculated the mean in the process of wrangling and get the data. Then, we described the data we used the median and the interquartile range** (**IQR).**

- Line 246-247: Please be careful with the extra spaces between words and “min-“

**Done. Thank you**

- Line 255: What does “The global median” mean? Please rewrite

**Done. Thank you**

- Lines 242 and 257-259: Authors state that the values were higher or lower, it is necessary to apply the corresponding statistical analysis in order to ascertain if the differences are statistically sound and to quantify the differences. This analysis would reinforce the results and the power of the paper.

**Thanks for your suggestion. In the Figure 1 (box plots) we only describe the pattern of the sample. From the point of view of Miguel Hernan or Rasmus Nielsen, we have described the aim of our study as just descriptive and does not intend to use inference.**

**Hernán, M. A., Hsu, J., & Healy, B. (2019). A second chance to get causal inference right: a classification of data science tasks. *Chance*, *32*(1), 42-49.**

**Nielsen, R. O., Simonsen, N. S., Casals, M., Stamatakis, E., & Mansournia, M. A. (2020). Methods matter and the ‘too much, too soon’theory (part 2): what is the goal of your sports injury research? Are you describing, predicting or drawing a causal inference?.**

- Table 2: remove the title of the table form the table, they should be apart

**Done. Thank you**

- Table 2: Remove quotation marks

**Done. Thank you**

- Table 2: The number of injuries was very low, besides medical attention were included (no time loss injuries) in comparison to other studies i.e. Ekstrand J, Hägglund M, Waldén M. Epidemiology of muscle injuries in professional football (soccer). Am J Sports Med. 2011;39(6):1226-32. doi: 10.1177/0363546510395879. This issue should be discussed accordingly
- Line 281: Authors state that TL injury incidence was higher in the professional players vs youth players (1.59 vs 1.34), however, due to the fact that there is no statistical analysis this cannot be corroborated. The same applies to other results i.e. 0.25 vs 0.20 (line 289) what is the real relevance of this? Are they really “different”?. A proper statistical analysis would help to this.

**As pointed above, we have described the pattern of the sample.**

DISCUSSION

- The discussion should start with an “introductory paragraph of the discussion section) including a summary paragraph and the objectives of the study.
- The discussion section is poor. Authors should discuss their results and explain them, giving reasons for it. In addition, they should compare to other studies.
- Paragraphs and sentences should follow a flow of results - ideas – comparison to other studies – discussion of the similarities/disparities….
- Practical applications: This paragraph should be rewritten in order to better clarify the real application of this study.
- Line 340: Please correct the sentence (professional and youth players?)

**Done. Thank you**

- Line 341-344: What is the reason for these sentences? It seems that this is an explanation for a result? Perhaps place it after the next paragraph?

**Done. Thank you**

- Line 345: The authors state that results of the external load was similar between professional and youth players; nevertheless, due to the low quality of the graphs it is not possible to see this results, there are no results written in the results section and there is no statistical analysis to confirm the similarities or the differences in the results.

**We have followed the Figure File Requirements (https://journals.plos.org/plosone/s/figures#loc-figure-preparation-checklist) of the Plos and we haven’t had any problems when submitting it. It may be due to OS systems.**

**The description of Figures 1 and 2 are not too long and we only report the general pattern because the main objective of the study is to explain the incidences.**

- Line 345-346: The authors explain their results but include an external reference (21). Please amend this incongruence

**Done. Thank you**

- Lines 353-363: check the format of the references and correct them. Check the English.

**Done. Thank you**

- Line 358: Difficult to understand “467 observed”

**Done. Thank you**

- Line 362. Check the verb tense. Also, link better both paragraphs.

**Done. Thank you**

- Line 368: This subtitle is not correct. Differences?

**Done. Thank you**

- Line 373: Correct the verb. Correct the term “what happens” it is very colloquial

**Done. Thank you**

- Lines 370-379: These are results; authors should avoid repeating results, unless it is necessary and discuss them, find the relevance and the meaning of the results and compare them to other authors.

**To our knowledge, it is the first time that such analysis is performed in elite footballers.**

- Lines 383-387: These are results again. The authors should mention the reasons for these results and the relevance

**Done. Thank you**

- Lines 388-391: There is an incongruence: the same training methodology leads to a difference in the injury incidence? Please explain

**Done. Thank you**

- Practical applications: Authors should rewrite these applications, in this version the applicability of the study is missing
- Conclusions: this paragraph is a summary of the results. Please rewrite the conclusions of the study

OVERALL COMMENTS

- The quality of the graphs is poor. It is very difficult to read the numbers and the letters
- There are many errors throughout the manuscript
- Check all the References: there are many mistakes, for example ref number 6, 11 (capital letters), 14 etc; remove [internet] and so on.

**Done. Thank you**
